# Detection of Pathogenic *Escherichia coli* and *Staphylococcus aureus* from Cattle and Pigs Slaughtered in Abattoirs in Vhembe District, South Africa

**DOI:** 10.1155/2015/195972

**Published:** 2015-02-24

**Authors:** Nicoline F. Tanih, Eunice Sekwadi, Roland N. Ndip, Pascal O. Bessong

**Affiliations:** ^1^HIV/AIDS & Global Health Research Programme, Department of Microbiology, University of Venda, Thohoyandou 0950, South Africa; ^2^Department of Microbiology and Parasitology, Faculty of Science, University of Buea, Buea, Cameroon; ^3^Department of Biochemistry and Microbiology, Faculty of Science and Agriculture, University of Fort Hare, Alice 5700, South Africa

## Abstract

Pathogenic food-borne bacteria have been associated with severe morbidity and mortality in humans and animals. This study was aimed at determining the prevalence of *Staphylococcus aureus, Salmonella* spp., and *Escherichia coli* present in cattle and pigs slaughtered in selected abattoirs in Vhembe District and at determining the susceptibility of the isolates to antibiotics. A total of 176 swab samples (28 cattle and 16 pigs) of the rump, flank, brisket, and neck of the animals were analyzed using standard microbiological methods. *E. coli* isolates were genotyped to detect pathogenic strains. Of the 176 samples, 104 (67.5%) were positive for *E. coli* and 50 (32.5%) for *S. aureus*. There was no statistically significant difference (*P* > 0.05) in the isolation rate from the different animal parts or abattoirs. Overall, 14/104 (13.46%) of the *E. coli* isolates were pathogenic strains which included enteropathogenic *E. coli* (EPEC) (*bfp*A) 1.9%, enterotoxigenic *E. coli* (ETEC) (*LT*) 3.8%, and enteroaggregative *E. coli* (EAEC) (*aaiC*) 7.6%. *E. coli* isolates were resistant (100%) to vancomycin and bacitracin. *S. aureus* (100%) were resistant to oxacillin and nalidixic acid. The presence of resistant strains of these bacteria in food of animal origin could serve as important vehicles transmitting these bacteria to humans. This finding is of epidemiological significance.

## 1. Introduction

Food-borne pathogens have been extensively incriminated worldwide as common causes of bacterial infections in humans with food animals serving as important reservoirs [1–3]. In industrialized countries microbiological food-borne illnesses were reported in up to 30% of the population [4]. The majority of morbidity and mortality related to food-borne infections are caused by bacterial agents [5–7]. Food poisoning is commonly manifested as diarrheal diseases which are often triggered either by toxin production by the microbe or by the host's reaction to the infection [5, 7, 8].

A number of pathogenic bacteria have been associated with food of animal origin; these include amongst others* Salmonella* spp.,* Campylobacter* spp.,* Staphylococcus aureus, Listeria monocytogenes, Clostridium perfringens, Clostridium botulinum, E. coli* 0157:H7, and enterohemorrhagic* E. coli* (EHEC) [1, 3, 9, 10]. Staphylococci are normal inhabitants of the skin and mucous membranes of animals and humans, and strains with pathogenic potential are known to cause diseases which range from simple abscesses and mastitis to the more severe toxic shock syndrome [11–13]. Mastitis in cattle has equally been associated with a number of microorganisms including* Escherichia coli* and* S. aureus* [13, 14].* Salmonella enterica* is a significant cause of morbidity and mortality in humans and animals, with contaminated food of animal origin, particularly meat products from cattle and pigs, being an important source of human infections [1, 2, 15].* Escherichia coli* occur as normal flora in the gastrointestinal tract of humans and animals. However, pathogenic* E. coli* strains have been reported to cause life threatening infections in humans worldwide [15, 16].

Antibiotic resistance remains a major challenge in human and animal health. Resistance is increasingly being recognized in pathogens isolated from food [1, 17–19]. Food contamination with antibiotic-resistant bacteria can therefore be a major threat to public health, as the antibiotic resistance determinants can be transferred to other bacteria of human clinical significance [20, 21]. Furthermore, transfer of these resistant bacteria to humans has significant public health implications by increasing the number of food-borne illnesses and the potential for treatment failure [21].

Food of animal origin could be contaminated from the farm, a situation which may be further compounded if the food is not properly handled during slaughtering and processing giving way for pathogens to multiply [22]. The conditions under which these foods are handled raise questions regarding their microbiological quality. Studies conducted in different countries to investigate the microbiological quality of food of animal origin reported the presence of potential human pathogens [1, 2]. In South Africa, a large proportion of the population relies on beef and pork as their source of protein which could expose them to infection if contaminated [3, 16]. Even though other studies have reported the health risk associated with consumption of such products, there is a paucity of studies on the microbiological quality of cattle and pig slaughtered in abattoirs in the Vhembe District of Limpopo Province. The present study was carried out to assess the microbiological quality of beef and pork slaughtered in this area in a bid to throw more light on the inherent risk associated with such foods.

## 2. Materials and Methods

### 2.1. Study Site Description

The study sites included Vygeboomdrift pig abattoir (A1), C-net (A2), Mukwevho (A3), and Shayandima (A4), all of which are found in the Vhembe District, Limpopo Province, South Africa. C-net, Mukwevho, and Shayandima are cattle abattoirs where people occasionally bring their personal cattle for slaughtering for occasions such as funerals, weddings, or family gatherings. Vygeboomdrift abattoir is a commercial abattoir where pigs are slaughtered.

### 2.2. Sample Collection

Samples were collected according to the method of Pearce and Bolton [24]. A total of 176 swabbed samples were collected from 28 cattle (8, 8, and 12 cattle from abattoirs 2, 3, and 4, resp.) and 16 pigs from abattoir 1. Four parts of each animal which included the neck, brisket, rump, and flank were sampled with sterile swab rinsing kit (containing 10 mL isotonic buffer rinse solution). Sample swabbing of the carcasses was performed after the removal of the gastrointestinal tract. The tip of the swab was moistened with rinse solution before swabbing the carcass. The area for swabbing was selected by using 100 cm^2^ sterile disposable plastic template (Analytical Diagnostics, USA). The swabbing was done 10x horizontally and 10x vertically at each site. The total areas sampled were 400 cm^2^ of each carcass. Samples were transported on ice and processed within 2 hours.

### 2.3. Microbial Analysis

#### 2.3.1. Isolation and Identification of Bacterial Pathogens

*Salmonella* spp. were identified using 1 mL meat rinsed solution mixed with 9 mL buffered peptone water (Oxoid) and incubated at 37°C for 24 hours. After incubation 1 mL of preenrichment broth was transferred into 9 mL of Rappaport-Vassiliadis (RV) soya peptone broth (Oxoid) and Brilliant green agar (Oxoid) plates and incubated at 37°C for 24 hours.

For identification of* S. aureus*, the swab from swab rinsing solution was spread-plated on mannitol salt agar (Oxoid) plates and incubated at 37°C for 24 hours. Staphylococci coagulase positive colonies were confirmed using Staphylase Test Kit (Oxoid). Yellow colonies which were Gram positive cocci in cluster, oxidase negative, and coagulase and catalase positive and which produced clots were recorded as* S. aureus* and maintained at −20°C in 20% glycerol brain heart infusion broth for further studies.* S. aureus* ATCC 25923 was used as a positive control.

*E. coli* was identified by pipetting 1 mL of rinsed solution in three test tubes each containing 9 mL Brilliant green broth (Oxoid) fitted with Durham tubes and incubated at 45°C for 48 hours. Tubes with gas bubbles in the Durham tubes were streaked on MacConkey agar (Oxoid) plate and incubated at 37°C for 24 hours. Suspected colonies were tested for indole production. Pink colonies on MacConkey media which were indole positive were considered positive for* E. coli* and maintained at −20°C in 20% glycerol brain heart infusion broth for further confirmation and characterization by PCR and antibiotic susceptibility testing.* E. coli* ATCC 25922 was used as a positive control.

Presumptive bacteria identification was based on colony pigmentation and Gram staining characteristics. Pure cultures were obtained by streaking a portion of an isolated colony on nutrient agar and incubated aerobically at 37°C for 24 h. All preliminary results were confirmed using the Microscan-Autoscan equipment (Siemens, Germany) following the manufacturer's instruction. Briefly, an inoculum of the bacterium was prepared in saline and transferred onto 96-well plates in an automated pattern. Reagents to supplement various reactions to aid identification of both Gram negative and positive bacteria were added to some of the wells and incubated at 37°C for 24 hours. Plates were read and results interpreted.

### 2.4. Molecular Identification of* E. coli* Using mPCR

#### 2.4.1. Extraction of DNA

*E. coli* cultures were revived by streaking on nutrient agar (Oxoid) and incubated at 37°C for 24 hours. Thereafter, 0.5 *μ*L of Triton X and 500 *μ*L of sterile distilled water were mixed with one colony in a 1.5 mL Eppendorf tube and mixed by vortexing for 5 seconds. The mixture was boiled in a water bath at 100°C for 20 minutes and centrifuged at 10000 rpm for 10 minutes. Five microlitres of the supernatant was used as DNA template for polymerase chain reaction.

#### 2.4.2. Multiplex Polymerase Chain Reaction (mPCR)

Multiplex polymerase chain reaction analysis of the targeted genes of interest was performed using DreamTaq DNA polymerase (Thermo Scientific, USA). For the amplification, five microlitres of DNA was added to 20 *μ*L of master mix containing 12.5 *μ*L of DreamTaq DNA polymerase (2X DreamTaq Green Buffer, dATP, dCTP, dGTP, and dTTP, 0.4 mM each, and 4 mM MgCl_2_) (Thermo Scientific, USA), 0.5 *μ*L (0.2 *μ*M) of respective oligonucleotide primers and the reaction volume was made up with nuclease free water. PCR was performed in a thermal cycler (Bio-Rad Laboratories, USA). The amplification cycles consisted of an initial DNA denaturation at 95°C for 15 min, followed by 35 cycles of denaturation at 94°C for 45 s, primer annealing at 55°C, for 45 s, extension at 68°C for 2 min, and a final single elongation at 72°C for 5 min. The primers used to amplify the targeted genes were as previously reported by Nguyen et al. [23] and are summarized in Table 1. Negative controls, substituting DNA template with ultrapure water (Sigma-Aldrich, UK), were included in all PCR runs. DNA extracted from* E. coli* ATCC 25922 was used as a positive control. Amplified DNA was resolved by 2% agarose gel electrophoresis and visualised under UV transillumination.

### 2.5. Antibiotic Susceptibility Testing

Antibiotic susceptibility testing was performed by the Kirby-Bauer disc-diffusion test, which conforms to the recommended standard of the Clinical and Laboratory Standards Institute (CLSI) as previously described by Nyenje et al. [25]. Briefly, an inoculum of each pure bacterial isolate was emulsified in 3 mL of sterile normal saline and the density adjusted to 0.5 McFarland standard. A sterile cotton swab was dipped into the standardized suspension of bacterial cultures and used to inoculate Mueller-Hinton agar (MHA) plates (Biotec, England), and the plates were allowed to dry. Antibiotic discs with the following drug contents ampicillin (10 *μ*g), bacitracin (10 *μ*g), erythromycin (15 *μ*g), oxytetracycline (30 *μ*g), streptomycin (10 *μ*g), cephalothin (30 *μ*g), nalidixic acid (30 *μ*g), gentamycin (10 *μ*g), vancomycin (30 *μ*g), and oxacillin (1 *μ*g) (Antibiotic Becton, Dickson and Company, Sparks, USA; Le Pont de Claix, France) were placed onto MHA plates. The plates were incubated at 37°C for 24 hours. The zone diameter was measured and results were interpreted based on CLSI [26]. The reference strains* E. coli* ATCC 25922 and* S. aureus* ATCC 25923 were used to verify the quality and accuracy of the testing procedure.

### 2.6. Statistical Analysis

Statistical analysis was performed using SPSS version 22. The chi-square test was used to compare rate of isolation of the various pathogens in beef and pork and the different animal parts sampled. Comparisons were also done among the abattoirs. Differences were considered significant at *P* < 0.05.

## 3. Results

### 3.1. Prevalence of Bacteria Pathogens in the Various Animal and Abattoir Types

Table 2 depicts the prevalence of pathogens investigated in the 176 samples examined. Overall, a high prevalence of 87.5% (154/176) was reported from the samples examined. Bacteria were isolated in all the abattoir types; the most prevalent bacteria were* E. coli* 67.5% (104/154), while* S. aureus* was 32.5% (50/154). No* Salmonella* was isolated in this study. Both* E. coli* and* S. aureus* were more prevalent in pork with percentages of 48.1% (50/104) and 40% (20/50), respectively, than in cattle across the different abattoirs. The isolation rate of the pathogens from cattle and pigs was however not statistically significant (*P* > 0.05). Overall, isolation rate of both organisms combined was highest from the neck samples 36.4% (56/154) followed by brisket 29.8% (46/154), flank 23.4% (36/154), and rump 10.4% (16/154) (Table 2).

Abattoirs 1-pork A1 and 2-beef A2 had the highest isolation rates of 45.45% (70/154) and 19.48% (30/154), respectively. The lowest isolation rate of 9.1% (14/154) was obtained in abattoir 3-beef A3 (Table 3). The isolation rate of the pathogens between the different abattoirs was not statistically significant (*P* > 0.05).

### 3.2. Prevalence of Pathogenic* E. coli*

Overall, pathogenic* E. coli* was detected in 13.46% (14/104) with 1.92% of* bfpA* (EPEC), 3.84% of* LT* (ETEC), and 7.69% of* aaiC* (EAEC) (Table 4). EIEC (enteroinvasive* E. coli*) was not detected in this study.

### 3.3. Antimicrobial Patterns

All* E. coli* isolates tested (100%) were susceptible to nalidixic acid, cephalothin, gentamycin, and ampicillin; 90% were susceptible to streptomycin. A hundred percent resistance was recorded for bacitracin and vancomycin, while resistances of 98%, 92%, and 5.7% were reported for oxacillin, erythromycin, and streptomycin, respectively. The rest showed either intermediate or total resistance to these antibiotics (Table 5). Of the 50* S. aureus* isolates, 100% susceptibility was recorded for cephalothin, gentamycin, ampicillin, streptomycin, vancomycin, and erythromycin, while 88% and 56% were recorded against oxytetracycline and bacitracin, respectively. On the other hand, 100% resistance was noted for nalidixic acid and oxacillin (Table 5). Multidrug resistance was not common in this study. Only two isolates were resistant to more than two antibiotics; this included oxacillin, nalidixic acid, oxytetracycline, and bacitracin.

## 4. Discussion

Foods contaminated with enteropathogenic bacteria are an important factor contributing to the high incidence of diarrhea in developing countries [27]. Pathogenic* E. coli*, nontyphoid* Salmonella* serovars, and* S. aureus* remain a potential threat to human health with beef, broiler chickens, and pork serving as possible sources of these organisms in the environment [2, 15, 16]. The clinical significance of these pathogens cannot be overemphasized. Pathogenic* E. coli* is recognized as an important pathogen in outbreaks of acute diarrhea especially in developing countries [7, 28, 29]. This study investigated the prevalence and antibiogram of these pathogens in a bid to provide baseline data for epidemiological surveillance.

Overall,* E. coli* 104/154 (67.5%) was the most detected pathogen followed by* S. aureus* 50/154 (32.5%). These findings corroborate those of other studies that equally reported a high prevalence of either* E. coli*,* S. aureus,* or both [19, 21, 22, 30]. Several studies have reported the presence of* E. coli* 0157:H7 in beef and pork carcasses [5, 15, 16, 31, 32]. However, our study focused on diarrheagenic* E. coli* pathotypes. Interestingly, some* E. coli* pathotypes were detected in this study. Rivas Palá and Sevilla [33] in their study also found* S. aureus* in 16.90% of meat samples.* Salmonella* was not detected in our study, a finding which is similar to that of Movassagh et al. [3] who did not also report* Salmonella* isolates in their study on beef carcasses. The high prevalence of these organisms in these animals could result from consumption of contaminated feed [2] or grazing plants that may have been contaminated through fertilization with untreated effluents or sludge. There is a high probability that the immediate environment of these animals was not endemic with* Salmonella*. However, our findings are contrary to other studies which reported the presence of* Salmonella* in beef and pork [1, 2, 15, 32, 34, 35]. Both organisms (*E. coli* and* S. aureus*) combined were more isolated from neck samples 36.4% (56/154). Our results tie with the finding of Pearce and Bolton [24] who reported a higher isolation rate of Enterobacteriaceae from the neck and shoulder regions of slaughtered animals in their study. The rump was the site with the least isolation rate 10.4% (16/154). This could be due to the fact that microorganism needs enough nutrients and oxygen to grow and multiple which could be absent in the rump given is made up of mostly muscles. Initial contamination of meat is likely to occur during slaughtering [36]. According to studies by Podpecan et al. [36] the presence of* S. aureus* in meat commonly indicates contamination that may be directly introduced by the hands of workers and contaminated equipment.

The rates of microbial contamination of abattoirs meat with* E. coli* and* S. aureus* in this study ranged from 6.5% to 32.5% in the different abattoirs. The pathogens were isolated more frequently from 1-pork A1 abattoir, though the difference was not statistically significant (*P* > 0.05). Worthy of note is the fact that 1-pork A1 abattoir is a pig abattoir. This finding may not be far from reality given that pigs are filthy compared to cattle. The organisms were also isolated in the other abattoirs in our study. The lowest isolation rate of 9.1% (14/154) was found in abattoir 3-beef A3. The sanitation level in this abattoir was seemingly better than the others and could explain this difference.

Of significance is the fact that 13.46% (14/104)* E. coli* strains isolated were positive for pathogenic* E. coli*. EAEC was the most detected pathotype with a prevalence of 7.69% (8/104) followed by ETEC 3.84% (4/104) and EPEC 1.92% (2/104). Enteroinvasive* E. coli* (EIEC) and EHEC were not detected in this study. The extremely high prevalence of nonpathogenic* E. coli* 92.3% (96/104) in this study may not be surprising. The majority of* E. coli* are harmless commensals of the mammalian gastrointestinal tract [37]. The presence of EAEC in this study is consistent with the works of Harrington et al. [37] who previously reported this organism in association with food-borne diseases. Also, EAEC is increasingly being reported as an emerging diarrheal pathogen worldwide [29]. The absence of STEC (EHEC) and EIEC in meat analysed in this study is interesting considering that other studies in different countries have reported* E. coli* 0157:H7 and other strains of STEC in abattoir meat, especially beef [38]. Specific pathotypes of* E. coli* have been reported to be prevalent in different geographical regions; hence our environment may be void of these pathotypes or they may exist in low prevalence.

The growing problem of antibiotic resistance has become a significant public health concern [19].* S*.* aureus* was 100% resistant to oxacillin and nalidixic acid in our study. This is in line with studies by Haimanot et al. [39] who reported* S. aureus* resistance of 90% to oxacillin. Also, Ateba et al. [13] reported high sensitivity of* S. aureus* to vancomycin similar to our findings and high resistance to ampicillin across the different farms studied in South Africa contrary to the findings of our study. Susceptibility to antibiotics changes with time and geographical location [1]. Also different antibiotic practices may account for such trends. Eight percent (4/50) of* S. aureus* were resistant to more than 3 antibiotics.

*E. coli* was susceptible to most of the antibiotics used in this study. Studies by Nontongana et al. [40] reported 98%* E. coli* resistance to ampicillin contrary to our result with 100% susceptibility to ampicillin. However, their study was focused on* E. coli* isolates from water sample. Our results are however similar to other studies that had previously reported resistances to one or more of the antibiotics that we recorded resistances to [13, 40, 41]. Resistance of these organisms to the antibiotics may be due to the frequent use of antibiotics in animal husbandry practices [13, 41], as most of these antibiotics are used both in human and in animal medicines. Resistant commensal bacteria of food animals such as pig and cattle may increase resistance of pathogenic bacteria in the intestinal tract of humans [42] causing disease that may be difficult to treat [43].

## 5. Conclusion

Our results indicate that cattle and pigs could serve as reservoirs of* S. aureus* and* E. coli* in the Limpopo Province of South Africa. These isolates were highly susceptible to a number of antibiotics which could form the basis for empiric treatment of infections caused by these pathogens in our environment. We are led to conclude that the absence of a statistically significant difference between different beef and pork carcasses from the different abattoirs might be due to the small sample size, even though some studies have not reported any statistical difference between the different animal carcasses. It may be that a large sample size must be studied to reveal statistically significant relations between the different animal carcasses.

## Figures and Tables

**Table 1:** Primer sequences used in multiplex PCR for detection of pathogenic *E. coli*.

*E. coli* type	Primer sequences	Product size in bp	References
ETEC (*LT*)			
ETEC 508F	5′-CACACGGAGCTCCTCAGTC-3′	508 bp	[23]
ETEC 508R	5′-CCCCCAGCCTAGCTTAGTTT-3′		
ETEC (*ST*)			
ETEC 147F	5′-GCTAAACCAGTAGAGGTCTTCAAAA-3′	147 bp	[23]
ETEC 147R	5′-CCCGGTACAGAGCAGGATTACAACA-3′		
EHEC (*Stx1*)			
EHEC 384F	5′-CAGTTAATGTGGTGGCGAAGG-3′	384 bp	[23]
EHEC 384R	5′-CACCAGACAATGTAACCGCTG-3′		
EHEC (*Stx2*)			
EHEC 584F	5′-ATCCTATTCCCGGGAGTTACG-3′	584 bp	[23]
EHEC 584R	5′-GCGTCATCGTATACACAGGAGC-3′		
EPEC (*eae*)			
EPEC 881F	5′-CCCGAATTCGGCACAAGCATAAGC-3′	881 bp	[23]
EPEC 881R	5′-CCCGGATCCGTCTCGCCAGTATTCG-3′		
EPEC (*bfpA*)			
EPEC 300F	5′-GGAAGTCAAATTCATGGGGGTAT-3′	300 bp	[23]
EPEC 300R	5′-GGAATCAGACGCAGACTGGTAGT-3′		
EIEC (*ipaH*)			
EIEC 423F	5′-TGGAAAAACTCAGTGCCTCT-3′	423 bp	[23]
EIEC 423R	5′-CCAGTCCGTAAATTCATTCT-3′		
EAEC (*aatA*)			
EAEC 650F	5′-CTGGCGAAAGACTGTATCAT-3′	650 bp	[23]
EAEC 650R	5′-CAATGTATAGAAATCCGCTGTT-3′		
EAEC (*aaic*)			
EAEC 215F	5′-ATTGTCCTCAGGCATTTCAC-3′	215 bp	[23]
EAEC 215R	5′-ACGACACCCCTGATAAACAA-3′		

**Table 2:** Bacteria distribution in the different parts of cattle and pig carcasses examined in the various abattoirs.

Abattoirs	Animal parts	*S. aureus *	*E. coli *	Pathogenic *E. coli *	Number of samples from each abattoir	Number of animals sampled
1-pork A1	P1 neck	10	16	2 (EAEC)		
	P2 brisket	2	12		64	16
	P3 flank	8	16			
	P4 rump	0	6			

2-beef A2	B1 neck	2	14			
	B2 brisket	8	8	2 (EAEC)	32	8
	B3 flank	0	4			
	B4 rump	0	4			

3-beef A3	B1 neck	2	0			
	B2 brisket	2	6		32	8
	B3 flank	4	0			
	B4 rump	2	0			

4-beef A4	B1 neck	4	8	2 (EAEC), 2 (ETEC)		
	B2 brisket	2	6	2 (EAEC), 2 (EPEC)	48	12
	B3 flank	2	2	2 (ETEC)		
	B4 rump	2	2			

Total		50	104	14		

**Table 3:** Bacteria distribution in cattle and pig carcasses examined in the various abattoirs.

Bacterial isolates	Abattoirs				Number (%) occurrence
	1-pork A1	2-beef A2	3-beef A3	4-beef A4	
	(*n* = 64)	(*n* = 32)	(*n* = 32)	(*n* = 48)	
*E. coli *	50 (32.5)	30 (19.5)	6 (3.89)	18 (11.7)	104 (67.5)
*S. aureus *	20 (12.98)	10 (6.5)	10 (6.5)	10 (6.5)	50 (32.6)

Total	70 (45.5)	40 (20.5)	16 (10.4)	28 (18.2)	154 (87.5)

**Table 4:** Prevalence of pathogenic *E. coli* from the various abattoirs.

Abattoirs	*E. coli *pathotypes and associated genes								Total *E. coli* *N*	Total (%) pathogenic
	EPEC		ETEC		EIEC	EAEC		EHEC		
	*bfpA *	*eae *	*LT *	*ST *	*ipaH *	*aatA *	*aaiC *	*Stx1/2 *		
Abattoir 1							2		54	2 (14.28%)
Abattoir 2							2		26	2
Abattoir 3									6	0 (0)
Abattoir 4	2		4				4		18	10

Total	2		4				8		104	14 (13.46)%

**Table 5:** Antimicrobial susceptibility profile of *S. aureus* and *E. coli* isolated from cattle and pigs carcasses.

Antibiotics	*E. coli* (*n* = 104) (%)			*S. aureus* (*n* = 50) (%)		
	R	I	S	R	1	S
Bacitracin	104 (100)	—	—	6 (12)	—	44 (88)
Erythromycin	96 (92.3)	2 (1.92%)	6 (5.7)	—	—	50 (100)
Vancomycin	104 (100)	—	—	—	—	50 (100)
Oxacillin	102 (98)	2 (1.92%)	—	50 (100)	—	—
Oxytetracycline	100 (96)	—	4 (3.8)	22 (44)	—	28 (56)
Nalidixic acid	—	—	104 (100)	50 (100)	—	—
Cephalothin	—	—	104 (100)	—	—	50 (100)
Gentamycin	—	—	104 (100)	—	—	50 (100)
Ampicillin	—	—	104 (100)	—	—	50 (100)
Streptomycin	6 (5.7)	4 (3.8%)	94 (90)	—	—	50 (100)
